# Building Cancer Control Capacity: a Mixed-Method Evaluation of the Research to Reality (R2R) Mentorship Program

**DOI:** 10.5888/pcd11.130275

**Published:** 2014-02-20

**Authors:** Michael Sanchez, E. Peyton Purcell, Joan S. Michie, Sophia P. Tsakraklides, Madeline La Porta, Cynthia Vinson

**Affiliations:** Author Affiliations: E. Peyton Purcell, Clinical Research Directorate/CMRP, SAIC-Frederick, Inc, Frederick National Laboratory for Cancer Research, Frederick, Maryland; Joan S. Michie, Sophia P. Tsakraklides, Westat, Rockville, Maryland; Madeline La Porta, MS1; Cynthia Vinson, National Cancer Institute, Rockville, Maryland.

## Abstract

In 2011, the National Cancer Institute launched the Research to Reality (R2R) Pilot Mentorship Program to enhance mentees’ core evidence-based public health (EBPH) competencies. In this article, we describe the program and its evaluation results and the program’s ability to improve participants’ EBPH competencies and appropriateness of program components. Program evaluation consisted of a pre/post program competency questionnaire and interviews with mentees, mentors, mentees’ supervisors, and program staff. Mentees reported the same or higher rating in every competency at end of the program, with average increase of 0.6 points on a 4-point scale; the greatest improvements were seen in policy development/program planning. Mentorship programs are a promising strategy to develop EBPH competencies, provide guidance, and disseminate and adapt evidence-based interventions within real-world context.

## Introduction

Significant advancements have been made in understanding, preventing, and treating cancer (1). However, evidence-based interventions (EBIs) are not fully integrated into routine practice (2). Common challenges include limited time to gather evidence, lack of skilled personnel, limited access to data, inadequate funding, and differing perspectives of what constitutes “evidence-based” (3,4).

Mentorship programs have been effective in disseminating EBIs in medicine (5,6) and have had some success in public health (7,8). In 2011, the National Cancer Institute (NCI) launched the Research to Reality (R2R) Pilot Mentorship Program (the Program) to enhance mentees’ core evidence-based public health (EBPH) competencies (9,10) through 3 primary program components: 1) mentorship and mentee projects, 2) training and support, and 3) online community platform (Figure). A complete description of the Program is available elsewhere (10).

**Figure:**
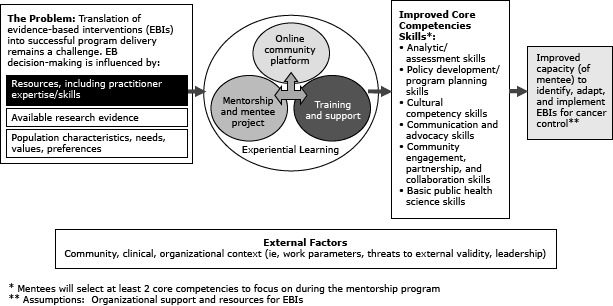
Research to Reality Pilot Mentorship Program logic model, National Cancer Institute, 2011. Source: Purcell et al (10).

**Table:** 

The figure depicts the framework for the Research to Reality (R2R) Mentorship Program showing 4 boxes that are connected with unidirectional arrows going from left to right.
Beginning on the left, there is a box outlining the problem that the program attempts to address. The problem is that translation of evidence-based interventions (EBIs) into successful program delivery remains a challenge. Evidence-based decision making is influenced by 1) resources, including practitioner expertise/skills; 2) available research evidence; and 3) population characteristics, needs, values, and preferences.
The first influencer listed (resources, including practitioner expertise) is highlighted in green, and there is an arrow from there to the second box in the diagram. This box is a large circle, entitled “Experiential Learning”; this large circle contains 3 smaller interconnected circles, each indicating 1 of the 3 components of the program: 1) online community platform, 2) mentorship and mentee project, and 3) training and support.
An arrow connects these components to the third box, which represents the intermediate outcome: Improved Core Competency Skills. The 6 competencies are: 1) analytic/assessment skills; 2) policy development/program planning skills; 3) cultural competency skills; 4) communication and advocacy skills; 5) community engagement, partnership, and collaboration skills; and 6) basic public health science skills. There is a note indicating that mentees will select at least 2 of the core competencies to focus on during the program.
An arrow points from the third box to the final box, which shows the connection between the intermediate outcome to the distal outcome, which is improved capacity (of mentee) to identify, adapt, and implement EBIs for cancer control. A note indicates that it is assumed that there will be organizational support and resources for EBIs. Listed at the bottom of the figure are the external factors: community, clinical, organizational context (ie, work parameters, threats to external validity, and leadership) that are present throughout the framework depicted.

The purpose of this article is to 1) describe the Program and report evaluation results and 2) discuss the Program’s ability to improve participants’ EBPH competencies (9,11) and the appropriateness of program components.

## Methods

In May 2011, NCI issued a call for mentee applications through the R2R website, listserv announcements, and relevant newsletters. Applicant eligibility included 1) full-time employment with an organization that addressed cancer control and prevention, 2) supervisor support to participate, 3) at least a master’s level public health training or 2 to 3 years equivalent training or experience, and 4) experience working with community organizations. Applicants were required to propose a project that was relevant to their current work and that addressed a comprehensive cancer control plan goal. NCI staff scored applications from 1 (noncompetitive) to 7 (excellent) on factors related to appropriateness of the applicant’s training, experience, and feasibility of project in the context of the Program. NCI interviewed the top 8 mentee candidates then selected and matched 6 mentees with mentors.

The evaluation included a preprogram and postprogram competency questionnaire, which assessed changes in EBPH competencies. Mentees rated their skills on a 4-point Likert scale (1 = no ability, 4 = expert) across 6 competency areas: 1) analytic/assessment; 2) policy development/program planning; 3) cultural competency; 4) basic public health science; 5) partnership, collaboration, and community engagement; and 6) advocacy and communication. Additionally, postprogram individual interviews were conducted with mentees, mentors, mentees’ supervisors, and Program staff.

Participation and satisfaction data for trainings, webinars, mentor–mentee activities, Web analytics, and mentees’ project deliverables were also reviewed. Because of the small number of participants, most data were qualitative. Mentees rated their preprogram and postprogram EBPH competency level, and changes in ratings were averaged across all mentees. Data for interviews and project deliverables were analyzed through content analysis; coding categories were directly derived from the text data. The codes of the 2 analysts were checked for reliability and consistency. The institutional review board at Westat conducted and approved the evaluation.

## Results

Mentees entered the Program from different organizations, level of experience, topic interests, and competency development needs (Table 1). The EBPH competency areas selected most frequently by mentees were analytic/assessment; partnership, collaboration, and community engagement; and advocacy and communication skills, each of which was chosen by two-thirds of the mentees (Table 2).

**Table 1:** Research to Reality Pilot Mentorship Program, Mentee Applicant and Mentee Characteristics, National Cancer Institute, 2011

Applicant Characteristic	All Applicants, No. (%) (n = 48)	Selected Mentees, No. (%) (n = 6)
**Sex**		
Female	42 (88)	5 (83)
Male	6 (13)	1 (17)
**Highest degree**		
Less than Bachelor's	1 (2)	—
Bachelor degree	6 (13)	2 (33)
Masters (non–public health)	13 (27)	2 (33)
Masters (public health)	23 (48)	2 (33)
PhD or MD	5 (10)	—
**Cancer Control Coalition experience**		
Yes, currently	28 (58)	6 (100)
Yes, in the past	3 (6)	—
No, never	17 (35)	—
**Organization type^a^**		
Academic	9 (19)	2 (33)
Cancer center	11 (23)	1 (17)
Clinical center	10 (21)	1 (17)
Government	6 (13)	2 (33)
State	4 (8)	2 (33)
Local (county/tribal)	2 (4)	
Other	12 (25)	—
**Portion of job that includes planning and/or implementing cancer control programs**		
All (>95%)	21 (44)	4 (67)
Most (65%–95%)	14 (29)	1 (17)
About half (35%–64%)	6 (13)	1 (17)
Some (<35%)	5 (10)	—
Don't know/Prefer not to answer	2 (4)	—
**Prioritized competencies^a^**		
Advocacy and communication skills	22 (46)	4 (67)
Assessment/analytic skills	22 (46)	4 (67)
Basic public health science skills	12 (25)	—
Cultural competency skills	14 (29)	1 (17)
Partnership, collaboration, and engagement skills	28 (58)	3 (50)
Policy development/program planning skills	36 (75)	4 (67)
**Project primary cancer topic**		
Breast cancer	5 (10)	—
Cancer health disparities	4 (8)	—
Cervical cancer	2 (4)	1 (17)
Clinical trials accrual	2 (4)	—
Colorectal cancer	7 (15)	2 (33)
Obesity, diet/nutrition, physical activity	4 (8)	1 (17)
Patient navigation	3 (6)	—
Sun safety/skin cancer	1 (2)	1 (17)
Survivorship	9 (19)	—
Tobacco control	4 (8)	1 (17)
Other	7 (15)	—

Abbreviation: —, no responses.

a Total may exceed 100% because applicants could give more than 1 response.

**Table 2:** Priority Competency Areas Selected by Mentees, Average Ratings on a 4-Point Scale of all Mentees at Program Completion, and Average Increase in Ratings From Program Initiation to Completion: Research to Reality Pilot Mentorship Program, National Cancer Institute, 2011

Competency	No. of Mentees Selecting This Area	Average Rating at Program Completion^a^	Average Increase in Ratings
1. Analytic/assessment	4	3.0	0.8
2. Policy development/program planning	3	3.1	1.0
3. Cultural competency	1	3.1	0.2
4. Public health science	0	3.2	0.6
5. Partnership, collaboration, and community engagement	4	3.3	0.8
6. Advocacy and communication	4	2.7	0.4

a Each competency was rated by participants on a 4-point scale (1 = no ability, 4 = expert).

For every skill assessed, mentees gave themselves the same or a higher rating at the end of the Program; the greatest change was seen in policy development/program planning (average 1.0 increase on a 4-point scale). Analytic/assessment skills and partnership, collaboration, and community engagement showed average increases of 0.8 points (Table 2).

All Program components were implemented and well-received by participants. Interviews showed that strong administrative support helped participants maintain focus but also provided some flexibility regarding timelines and deliverables that helped with addressing challenges mentees encountered in planning and conducting their projects. Except for the website, the Program’s main platform, all other Program components were valued by most of the participants. Although participants praised the website’s functionality, they made limited use of it, with lack of time frequently mentioned as a primary cause for underuse. Components mentioned by 2 or more mentees were their mentee–mentor relationship, cohort relationship, trainings, and projects. Mentors valued most the interactions among participants, especially the mentee–mentor pairs, and communication and webinars. Several mentors also mentioned the site visit, project, and trainings. Additionally, all mentees considered the training webinars to be helpful and effective, and all mentees and mentors thought programmatic support was adequate.

Mentees reported improved skills and knowledge in project management, building partnerships, navigating politics, adapting EBIs and watching for fidelity, assessment and analytical skills, manuscript writing, and making presentations. All of these accomplishments occurred despite challenges, such as workplace and life changes and loss of project funding.

## Discussion

To our knowledge, there are no published studies evaluating mentorship programs as a strategy to improve competencies needed to integrate EBIs into public health practice. This evaluation was intended to describe the Program’s ability to improve participants’ EBPH competencies and the appropriateness of Program components designed to build capacity of cancer control practitioners to navigate “real world” context.

Our evaluation resulted in 3 noteworthy findings. First, all Program components were implemented, and most were valued by participants, including the mentor–mentee relationship, training webinars, and site visit. Second, mentees were able to negotiate the “real world” context that affects the conduct of EBIs, including project management, building partnerships, navigating politics, and adapting EBIs despite multiple challenges encountered. However, this growth did not extend to mentees’ organizations. Additional efforts to disseminate mentees’ lessons learned in their organizations should be explored. Finally, mentees reported the same or a higher rating for every EBPH competency assessed at the end of the Program, with an average increase of 0.63 points on a 4-point scale.

Although our evaluation was limited in size, the diversity of participants and their related projects indicates that a mentorship program is a feasible strategy across multiple settings, trainings, and contexts. A strength of our pilot program was its ability to remotely integrate evidence-based resources, interactive Web tools and trainings, and mentorship to assist cancer control practitioners with adapting and implementing EBIs to local context. Another strength was the use of multiple data sources and triangulation, which strengthened the evaluation findings. 

Our findings show that mentorship programs have great promise as an effective means to develop EBPH competencies for cancer control practitioners and provide guidance and technical assistance with adapting EBIs to “real world” local settings. Further research is warranted to replicate these results on a larger scale and in comparison with other strategies.
